# A Lactate Fermentation Mutant of *Toxoplasma* Stimulates Protective Immunity Against Acute and Chronic Toxoplasmosis

**DOI:** 10.3389/fimmu.2018.01814

**Published:** 2018-08-10

**Authors:** Ningbo Xia, Taifang Zhou, Xiaohan Liang, Shu Ye, Pengfei Zhao, Jichao Yang, Yanqin Zhou, Junlong Zhao, Bang Shen

**Affiliations:** ^1^State Key Laboratory of Agricultural Microbiology, Huazhong Agricultural University, Wuhan, China; ^2^Hubei Cooperative Innovation Center for Sustainable Pig Production, Wuhan, China; ^3^Key Laboratory of Preventive Medicine in Hubei Province, Wuhan, China

**Keywords:** *Toxoplasma*, lactate dehydrogenase, live vaccine, toxoplasmosis, cellular immunity

## Abstract

*Toxoplasma gondii* is an important zoonotic pathogen infecting one-third of the world’s population and numerous animals, causing significant healthcare burden and socioeconomic problems. Vaccination is an efficient way to reduce global sero-prevalence, however, ideal vaccines are not yet available. We recently discovered that the *Toxoplasma* mutant lacking both lactate dehydrogenases *LDH1* and *LDH2* (*Δldh*) grew well *in vitro* but was unable to propagate in mice, making it a good live vaccine candidate. Here, we tested the protection efficacy of ME49 *Δldh* using a mouse model. Vaccinated mice were efficiently protected from the lethal challenge of a variety of wild-type strains, including type 1 strain RH, type 2 strain ME49, type 3 strain VEG, and a field isolate of Chinese 1. The protection efficacies of a single vaccination were nearly 100% for most cases and it worked well against the challenges of both tachyzoites and tissue cysts. Re-challenging parasites were unable to propagate in vaccinated mice, nor did they make tissue cysts. High levels of *Toxoplasma*-specific IgG were produced 30 days after immunization and stayed high during the whole tests (at least 125 days). However, passive immunization of naïve mice with sera from vaccinated mice did reduce parasite propagation, but the overall protection against parasite infections was rather limited. On the other hand, *Δldh* immunization evoked elevated levels of Th1 cytokines like INF-γ and IL-12, at early time points. In addition, splenocytes extracted from immunized mice were able to induce quick and robust INF-γ and other pro-inflammatory cytokine production upon *T. gondii* antigen stimulation. Together these results suggest that cellular immune responses are the main contributors to the protective immunity elicited by *Δldh* vaccination, and humoral immunity also contributes partially. We also generated uracil auxotrophic mutants in ME49 and compared their immune protection efficiencies to the *Δldh* mutants. The results showed that these two types of mutants have similar properties as live vaccine candidates. Taken together, these results suggest that mutants lacking LDH were severely attenuated in virulence but were able to induce strong anti-toxoplasma immune responses, therefore are good candidates for live vaccines.

## Introduction

*Toxoplasma gondii* is an obligate intracellular parasite that infects all warm-blooded animals and humans (1). Generally, its infection in healthy people causes no or mild flu-like symptoms, thus most of the infections are not noticed. However, in susceptible pregnant women, *T. gondii* infection may have severe consequences such as abortion, neonatal death, congenital defects, and mental retardation of delivered babies (2, 3). In addition, it is also a high risk for individuals with compromised immune functions, such as AIDS and organ transplant patients (2). Due to the broad host range, a variety of agricultural important animals such as pigs and sheep are constantly challenged by *T. gondii*, causing substantial economic losses and public health problems (2, 4, 5). Folic acid metabolism inhibitors such as pyrimethamine and sulfadiazine are commonly used to treat toxoplasmosis but they do not work on chronic infections (6).

The control of *Toxoplasma* infection is rather difficult, one reason is that it has complex life cycle and multiple routes of transmission (7, 8). Cats are the definitive hosts of *T. gondii* and the oocysts shed by cats are thought to be a key source of human and animal infections (9). In addition, *T. gondii* can be transmitted between intermediate hosts through predation. Most of the *Toxoplasma* infection cases belong to chronic infection, where the parasites are encysted in muscles and central nerve system (called tissue cysts) of infected animals lifelong (7). Ingestion of raw or undercooked meat from such animals represents another important route of transmitting the parasites to humans and animals (2, 5). As mentioned above, encysted parasites at the chronic infection stage are resistant to most of the current therapeutics. Another challenge to the control of toxoplasmosis is the complex population structure of *T. gondii* strains. North America and Europe are dominated by three clonal strains (type I, II, and III), which display different acute virulence in mice (10, 11). However, in other parts of the world, the strains are much more diverse, particularly in South America (12, 13). A recent study demonstrated that genetically distinct strains may be able to superinfect the same host, indicating the lack of sufficient cross protection from immunization with one single strain (14). This study had important highlights for the design of *Toxoplasma* vaccines, particularly whole parasite-based vaccines.

Scientists have done tremendous amount of work to pursue an ideal vaccine against *T. gondii*. The first generation of vaccines contained killed parasites, or native antigens derived from soluble or secretory proteins of cultured tachyzoites. These vaccines only provided limited protection against further infections (15, 16). Then when the recombinant DNA technology became available, a variety of subunit vaccines were tried, mainly using surface or secretory proteins such as SAG1 and MIC2 (17–20). These recombinant protein or vector-based subunit vaccines were safer and easier to make than native antigen-based vaccines, however, they did not provide sufficient protection either [reviewed in Ref. (21, 22)]. The strategy that holds the most promise for a good *Toxoplasma* vaccine seems to be live attenuated vaccines. Currently there is one commercial vaccine (Toxovax^®^) available, which is derived from the S48 strain originally isolated from an aborted lamb and licensed for use to avoid congenital toxoplasmosis in ewes (23). The exact mechanisms of Toxovax^®^ as a vaccine are not well understood, but thought to be linked to its inability to form cysts or oocysts to complete the life cycle (24). Tachyzoites can be cleared efficiently by hosts’ immunity, therefore mutants defective in cyst formation have the potential to be vaccines. Encouraged by the success of Toxovax^®^ and to design safer live vaccines, scientists turned to genetically modified parasites. Among these, uracil auxotroph mutants defective in *de novo* UMP (uridine 5′-monophosphate) synthesis are promising (25–27). Mutants with inactivated *CPSII* or *OMPDC* grew well *in vitro* in the presence of extra uracil (25–27), but were unable to establish acute infection in animals, therefore were severely attenuated. These mutants were extensively studied in mice and displayed great potential to be good vaccines, but still need to be tested in other animals like pigs, sheep, and cats.

We recently discovered that *T. gondii* mutants with both lactate dehydrogenase genes deleted (*Δldh*) grew robustly *in vitro* but failed to propagate *in vivo* (28), very similar to the uracil auxotroph mutants. The reason for this growth difference is that, under *in vivo* conditions when oxygen is limited, lactate fermentation becomes a key energy supply pathway to support parasite replication. By contrast, oxidative phosphorylation alone provides enough energy for parasite reproduction *in vitro* when oxygen is rich. As such, *Δldh* mutant was greatly attenuated *in vivo*, even in immune-deficient animals (28). In this study, we set to analyze the protective immunity of the *Δldh* mutant as a vaccine using the mouse model. The results showed that vaccination of mice with tachyzoites of this mutant induced efficient protection against the challenge of a variety of strains.

## Results

### Tissue Cysts Derived From ME49 *Δldh* Were Severely Attenuated in Mice

**Figure 1:**
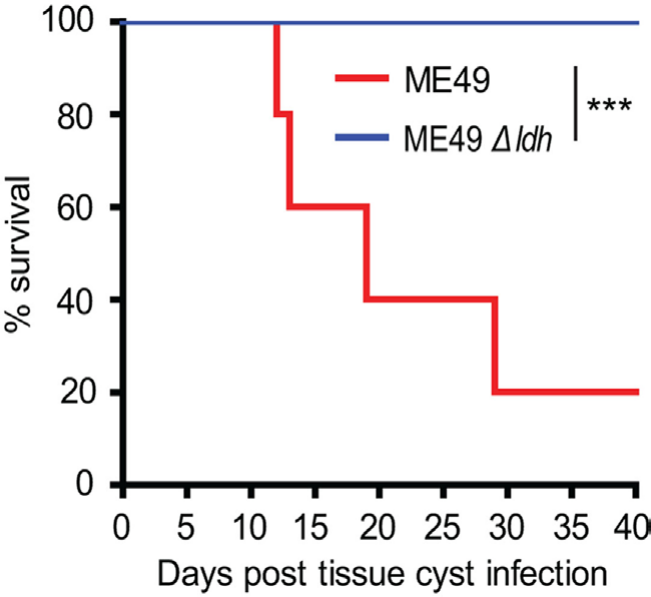
Virulence tests of *Toxoplasma* cysts in mice. ICR mice were infected with brain cysts of ME49 or ME49 *Δldh* (50 cysts/mouse, *n* = 5 mice for ME49 strain and *n* = 7 mice for ME49 *Δldh* strain) by oral administration and their survival was monitored for 40 days. ***p < 0.001, Gehan–Breslow–Wilcoxon test.

Our previous study reported that the virulence of ME49 *Δldh* tachyzoites was significantly (>640-fold) reduced compared to that of the wild-type (WT) strain ME49, no mortality was detected even at the infection dose of 3.2 × 10^4^ tachyzoites/mouse (28). However, we did observe that many of the *Δldh* tachyzoites infected mice formed brain cysts, although the amount was much less than that of WT parasites infected mice. To see whether the residual amount of cysts in *Δldh* infected mice have normal virulence, 50 cysts derived from WT or *Δldh* parasites were used to infect ICR mice by oral administration, and the survival of infected mice was monitored for 40 days. The results showed that 50 cysts of ME49 killed 80% of the mice. However, none of the *Δldh* cyst infected mice died (Figure 1), nor did they show any obvious clinical symptoms, suggesting that the virulence of the *Δldh* cysts was also significantly attenuated, just like the tachyzoites of this strain.

### *Δldh* Vaccination Elicited Strong Protective Immunity Against Tachyzoites Infection

**Figure 2:**
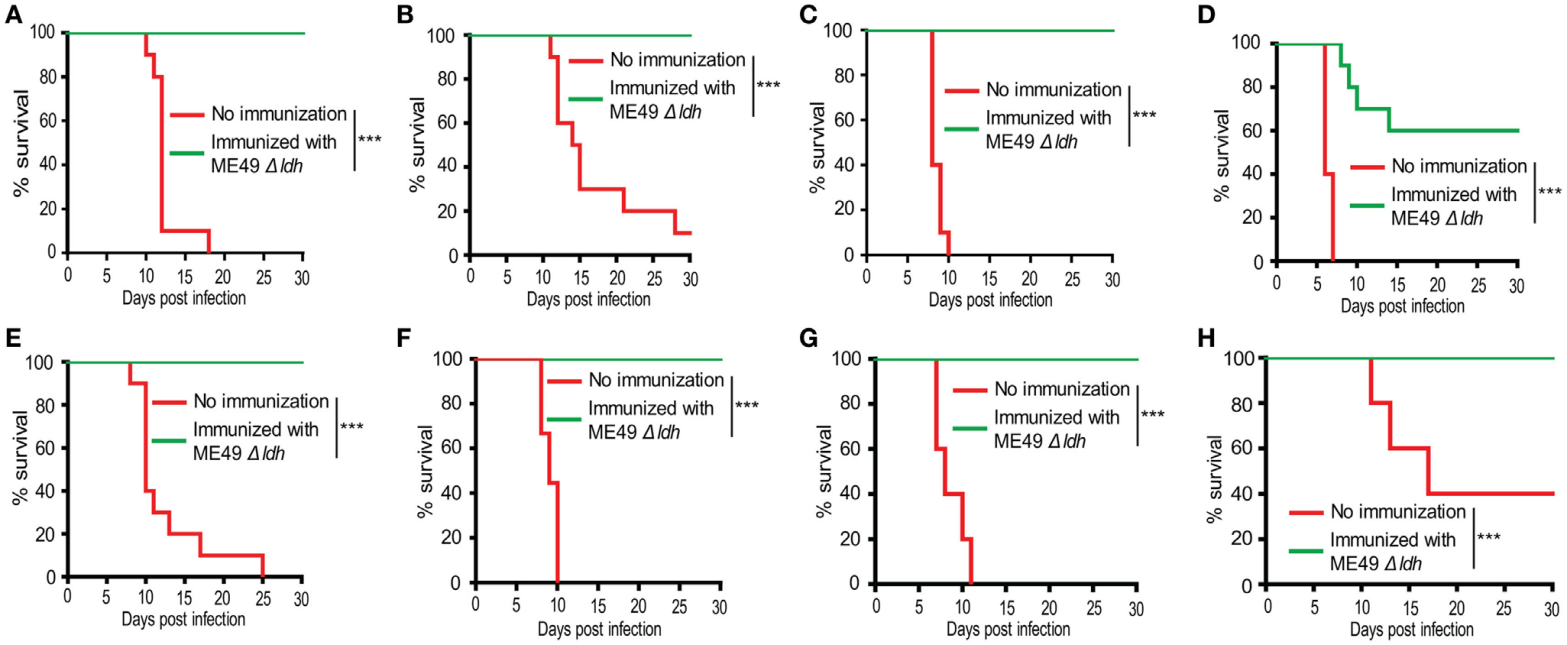
*Δldh* parasite immunization protected mice from *Toxoplasma gondii* tachyzoites infection. ICR mice were pre-immunized with 10^4^ tachyzoites of the ME49 *Δldh* mutant. **(A–C)** 30 days post-immunization, they were challenged with 10^4^ tachyzoites of the ME49 **(A)**, VEG **(B)**, or C7719 **(C)** strains (10 mice for each strain) by intraperitoneal injection and their survival was monitored for another 30 days. **(D–F)** 75 days post-immunization, 10^4^ tachyzoites of the RH *Δhxgprt* **(D)**, ME49 **(E)**, C7719 **(F)** strains were used to challenge the mice and their survival was monitored for another 30 days. **(G,H)** 125 days post-immunization, 10^4^ tachyzoites of the ME49 **(G)**, VEG **(H)** strains were used to challenge the mice and their survival was monitored for another 30 days. Non-immunized mice were included as control. ***p < 0.001, Gehan–Breslow–Wilcoxon tests.

The *Δldh* mutant grew robustly *in vitro* but was unable to propagate *in vivo*, this unique property makes it a good vaccine candidate (28). To check this possibility and assess the immune protection offered by *Δldh* vaccination, ICR mice were first immunized with ME49 *Δldh* by intraperitoneal injection (10^4^ tachyzoites/mouse). Thirty days later, they were challenged with 10^4^ tachyzoites of the type 2 strain ME49, type 3 strain VEG, or Chinese 1 strain C7719. Subsequently survival of the mice was monitored for another 30 days. The mortality rates for non-immunized mice were 100% upon the infection of ME49 and C7719, whereas VEG caused 90% mortality. However, for *Δldh*-vaccinated mice, the survival rates were 100% upon the challenge of all three strains and no obvious symptoms were observed during the 30-day infection period (Figures 2A–C). These results indicate that *Δldh* vaccination is able to offer efficient protection against lethal parasite infections.

The above results showed that 30 days post *Δldh* vaccination, mice were well protected. To see whether *Δldh* vaccination provides longer time protective immunity, mice were vaccinated with ME49 *Δldh* and 75 days later, they were challenged with type 1 strain RH *Δhxgprt*, type 2 strain ME49, or Chinese 1 strain C7719. The survival rates of immunized mice that were re-infected with ME49 or C7719 were 100% (Figures 2E,F), similar to the above infections 30 days post-vaccination. However, if challenged with the most virulent type 1 strain RH *Δhxgprt*, only 60% of the immunized mice survived (Figure 2D). As will be discussed below, the protection against RH infection was also 100% if the challenge occurred 20 days after immunization. To examine the efficiency of even longer time protection, ME49 *Δldh* vaccinated mice were challenged with WT parasites 125 days post-vaccination. The results showed that the immune protection against ME49 and VEG challenge was still 100% (Figures 2G,H). Together, these results suggest that ME49 *Δldh* immunization can provide both short- and long-term protection against strains with intermediate virulence, but the protection against strongly virulent strains is limited to a short time after vaccination and then decreased gradually.

### *Δldh* Immunization Stimulated Protective Immunity Against Bradyzoites Infection

**Figure 3:**
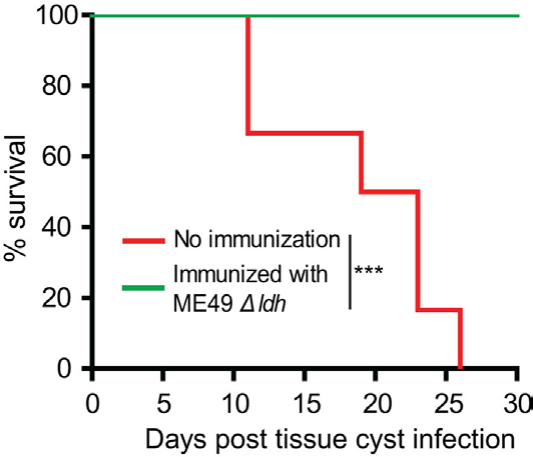
*Δldh* mutant vaccination protected mice from *Toxoplasma gondii* cysts infection. ICR mice were pre-immunized with ME49 *Δldh* as in Figure 2, 30 days post-immunization they were challenged with 50 fresh brain cysts of the ME49 strain and their survival was monitored for another 30 days (*n* = 10 mice for each strain). Non-immunized mice were included as control. ***p < 0.001, Gehan–Breslow–Wilcoxon test.

A significant portion of *T. gondii* infections were acquired through ingestion of tissue cysts that contain bradyzoites. In order to check whether *Δldh* vaccination provided protection against bradyzoites infection, ICR mice were first immunized with 10^4^ tachyzoites of ME49 *Δldh*. Thirty days after immunization, they were orally challenged with 50 cysts of the ME49 strain. The results show that 100% of *Δldh*-immunized mice survived the cyst challenging, whereas all naïve mice died of this infection (Figure 3). These results suggest that *Δldh* strain also elicited protective immunity against bradyzoite infection.

### *Δldh* Immunization Blocked Cyst Formation From New Infections

**Figure 4:**
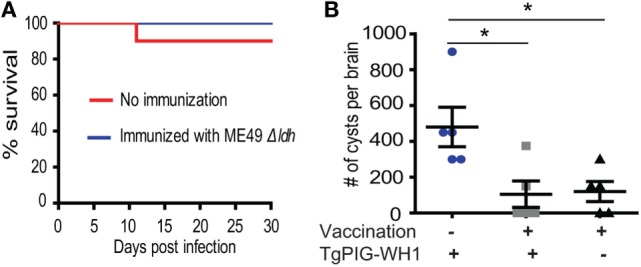
*Δldh* mutant immunization prevented cyst formation from further infections. **(A)** ICR mice were pre-immunized with ME49 *Δldh* as in Figure 2 and 30 days later challenged with 10^4^ tachyzoites of TgPIG-WH1. Non-immunized mice were included as control and survival of mice were monitored for another 30 days (*n* = 10 mice for each strain). **(B)** Cyst loads in the brains of non-immunized but TgPIG-WH1 challenged mice, immunized and TgPIG-WH1 challenged mice, and *Δldh* immunized but not TgPIG-WH1 challenged mice [not shown in **(A)**]. The mice survived at day 30 in **(A)** were sacrificed, and the number of *Toxoplasma* cysts in the brain homogenate was determined by DBA-FITC staining and fluorescent microscopy. Data from the analysis of five mice in each group were graphed, *p < 0.05, Student’s *t*-test.

One caveat of chemotherapies against acute toxoplasmosis is that the drugs may drive the parasites to slowly replicating bradyzoites to form tissue cysts, which lead to lifelong chronic infection. To examine whether the *Δldh* mutant vaccination could provide protective immunity against chronic *T. gondii* infection, the cyst forming competent but less virulent (compared with ME49) strain TgPIG-WH1 (genotyped as ToxoDB #3) was used to challenge the vaccinated ICR mice. As shown in Figure 4A, at the infection dose of 10^4^ tachyzoites/mouse through intraperitoneal injection, TgPIG-WH1 killed only 10% of the non-immunized mice, consistent with its reduced virulence. Thirty days post TgPIG-WH1 challenge, survived mice were sacrificed and the numbers of tissue cysts in the brains were determined by DBA-FITC staining, and compared to that of vaccinated but not re-challenged, as well as non-immunized but TgPIG-WH1 infected mice. The results in Figure 4B showed that infection of naïve mice by TgPIG-WH1 resulted in about 500 cysts per brain on average. However, cyst number in *Δldh* immunized and subsequently TgPIG-WH1 infected mice was reduced to around 100, which was very similar to that of vaccinated but not re-challenged mice, suggesting that they were probably derived from vaccination but not TgPIG-WH1 infection. Together, these results indicate that *Δldh* immunization also provides protective immunity against chronic toxoplasmosis.

### *Δldh* Vaccinated Mice Were Able to Clear Challenging Parasites Rapidly

**Figure 5:**
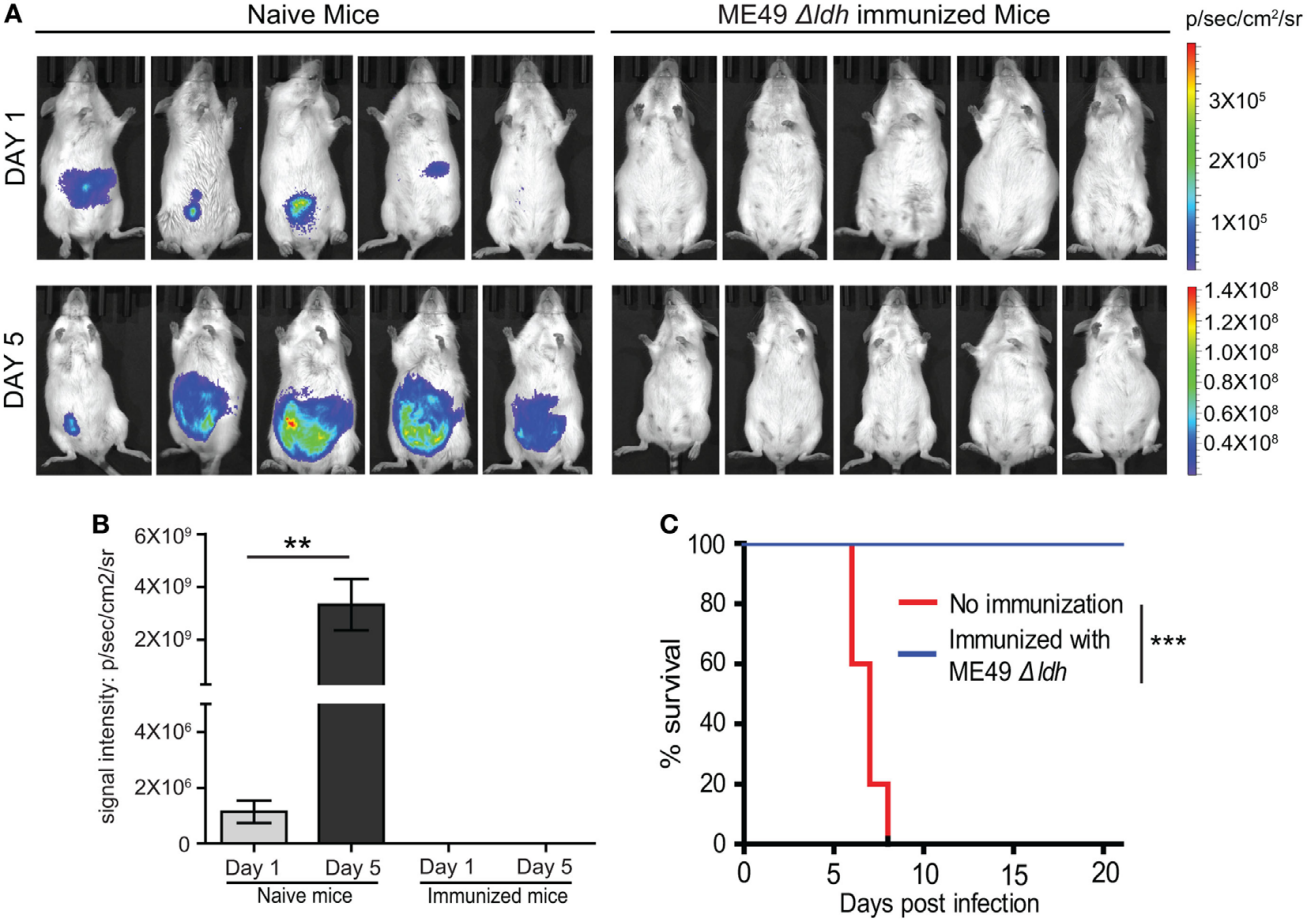
*Δldh* parasite vaccination elicited rapid clearance of challenging parasites. **(A)** Naïve or pre-immunized mice were challenged with 10^4^ tachyzoites of RH-Luc (five mice for each group). Parasite loads in mice 1 and 5 days post RH-Luc infection were analyzed by live animal imaging on the IVIS Spectra system. p/sec/cm^2^/sr: photons per second per cm^2^ per steradian. **(B)** Total bioluminescent signals of mice from **(A)** were calculated and graphed, **p < 0.01, Student’s *t*-test. **(C)** Survival curves of naïve or *Δldh* immunized mice infected with RH-Luc, ***p < 0.001, Gehan–Breslow–Wilcoxon test.

The above results demonstrated that vaccinating mice with ME49 *Δldh* provided efficient protection against both acute and chronic toxoplasmosis. To further examine the fate of challenging parasites, the propagation dynamics of a luciferase expressing strain RH-Luc in vaccinated mice were determined by bioluminescent imaging. In naïve mice, RH-Luc infection resulted in rapid replication of the parasites. At the infection dose of 10^4^ tachyzoites/mouse, luminescent signals were detectable just 1 day after infection and increased over 1,000-fold 5 days post-infection (Figures 5A,B). However, in the vaccinated mice, luminescent signals were not detectable at both time points, indicating little propagation and probably rapid clearance of the challenging RH-Luc (Figures 5A,B). It should be noted that in this experiment, RH-Luc infection was performed 30 days after *Δldh* immunization. We also followed the survival of these RH-Luc infected mice for 20 days, all naïve mice died within 8 days but all vaccinated mice survived with no symptoms (Figure 5C). These results suggest that, at least within a limited time period, *Δldh* immunization enables rapid clearance of the challenging parasites, which explains its efficient immune protection.

### *Δldh* Vaccination Significantly Increased the Levels of Pro-Inflammatory Cytokines and *T. gondii* Specific IgG Antibodies

**Figure 6:**
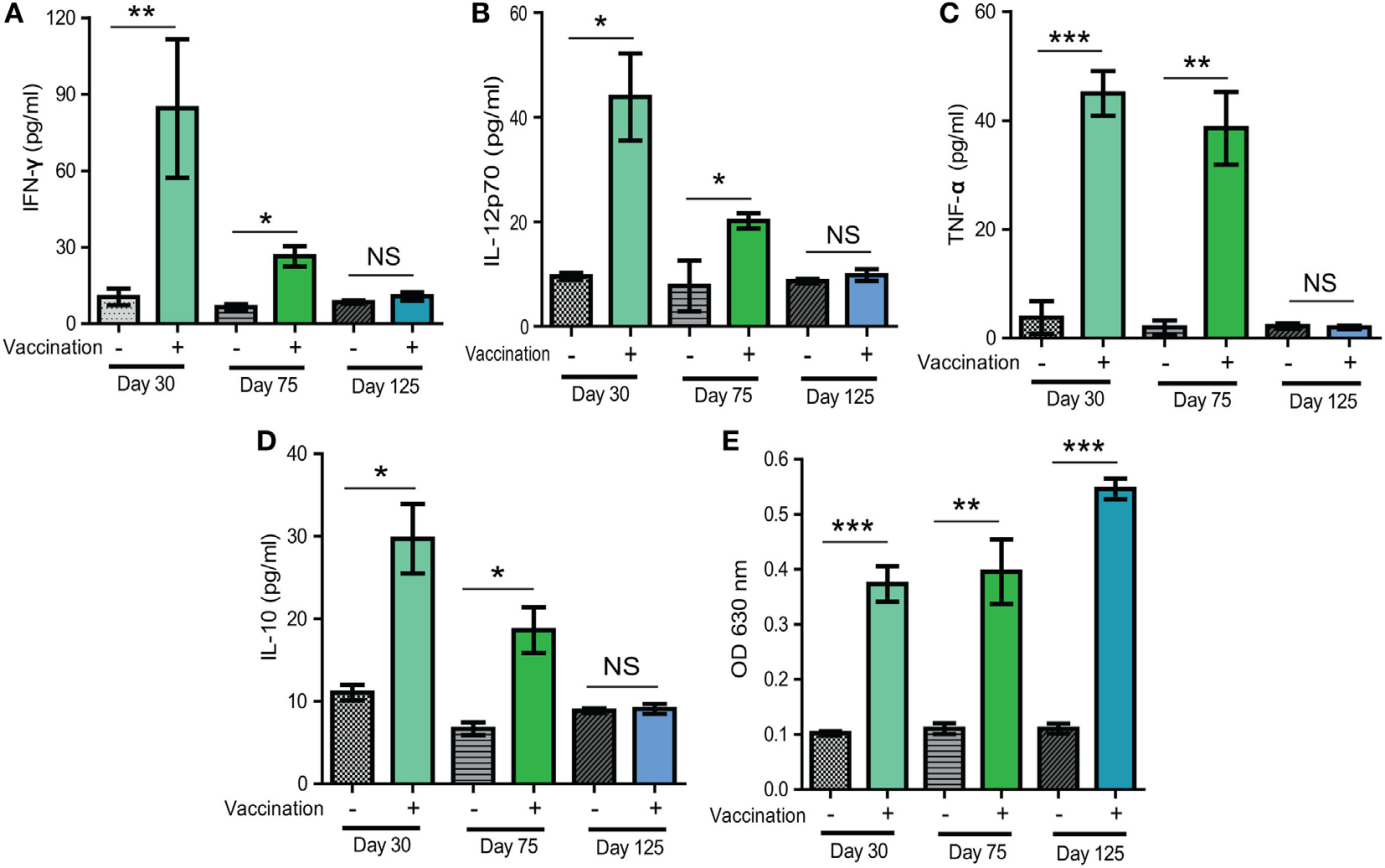
Levels of selected cytokines and *Toxoplasma* specific IgG in sera of mice 30, 75, and 125 days after *Δldh* immunization. **(A–D)** Levels of indicated cytokines measured by ELISA. **(E)** Relative levels of *Toxoplasma* specific IgG, as determined by indirect ELISA. Sera from naïve mice were used as controls and three mice from each group were analyzed. *p < 0.05, **p < 0.01, ***p < 0.001, NS: not significant, Student’s *t*-test.

To estimate the potential mechanisms of immune protection offered by *Δldh* vaccination, we first examined the cytokine level changes upon vaccination. Sera samples obtained from vaccinated mice 30, 75, or 125 days post-vaccination were subject to ELISA analysis to check the levels of IFN-γ, IL-12, IL-10, and TNF-α. The IL-12–IFN-γ axis is key to activate cellular immune clearance of *T. gondii*. Their levels, as well as the levels of another pro-inflammatory cytokine TNF-α, increased significantly both 30 and 75 days post-vaccination, compared to control mice (Figures 6A–C). However, the levels at day 75 were lower than that at day 30, which was likely caused by the activation of anti-inflammatory response, as evidenced by high levels of IL-10 in vaccinated mice 30 days post-infection (Figure 6D). At 125 days post-infection, the levels of all cytokines were back to normal as in naïve mice (Figures 6A–D). We also measured the *T. gondii* specific IgG levels and found that ME49 *Δldh* vaccination induced high levels of IgG 30, 75, and 125 days post-immunization (Figure 6E). Although it looked like there was an increase in *T. gondii* specific IgG levels over time, the differences were not statistically significant (Figure 6E). The relatively stable levels of parasite-specific IgG were in contrast to the changes of cytokine levels. These results, along with the above-described short- and long-term protective immunity (Figure 2), suggest that *Δldh* immunization probably stimulated both humoral and cell-mediated immune responses to control secondary infections.

### Passive Immunization With the Sera of *Δldh*-Vaccinated Mice Provided Partial Protection Against *T. gondii* Infection

**Figure 7:**
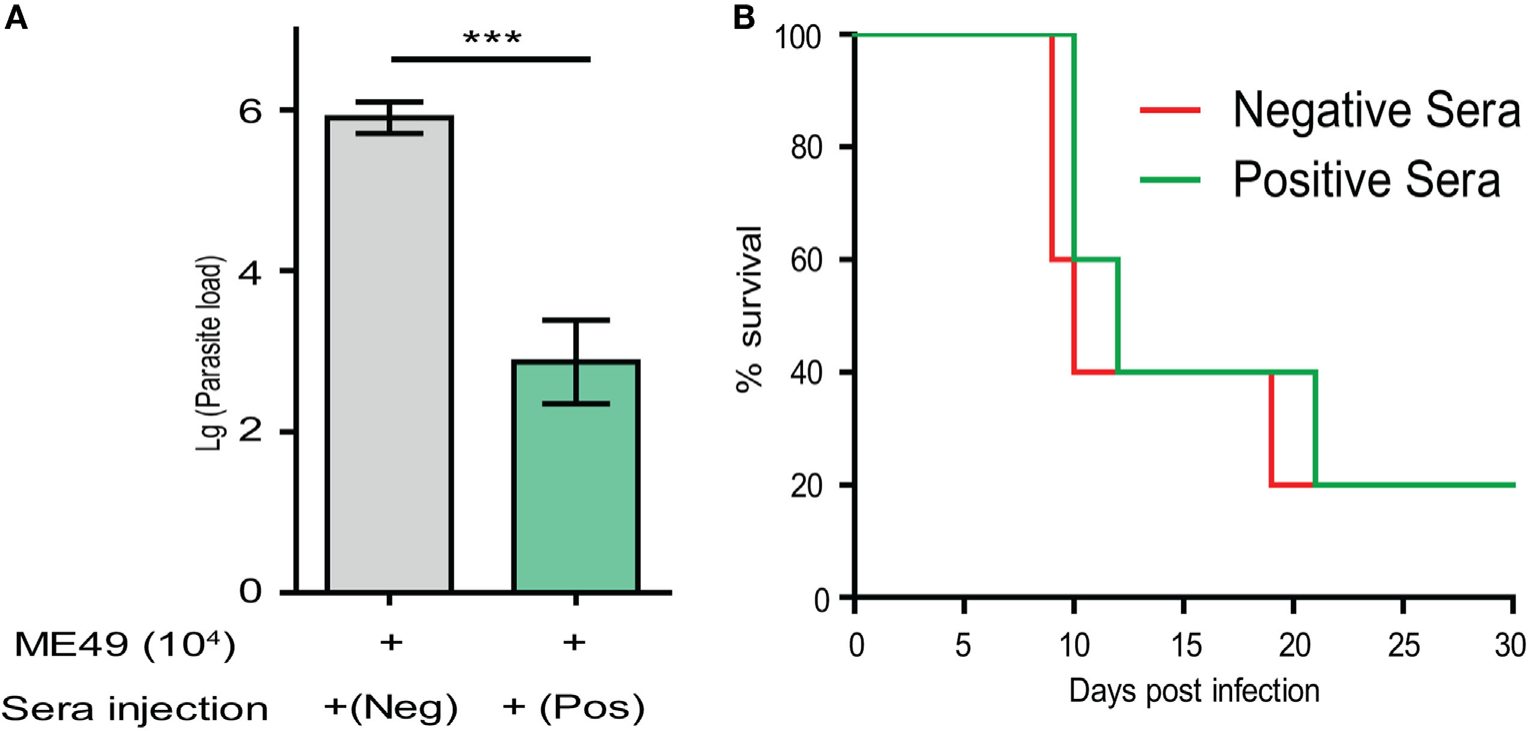
Passive immunization using the sera from *Δldh*-vaccinated mice provided partial protection against parasite infection. **(A)** ICR mice (*n* = 3 for each group) were injected with 10^4^ tachyzoites of ME49 by intraperitoneal injection. At day 0 and day 3 post-infection, mice were treated with positive sera collected from the ME49 *Δldh*-vaccinated mice (160 days post-vaccination), or negative serum from naïve mice by tail intravenous injection. Parasite loads in peritoneal fluids 7 days post-infection were estimated by quantitative PCR. ***p < 0.0001, Student’s *t*-test. **(B)** Survival of passively immunized mice.

Above results demonstrated that high levels of *T. gondii* specific IgG were produced in *Δldh*-vaccinated mice. To estimate the contribution of such antisera in restricting further parasite infection, naïve mice were infected with the WT strain ME49 through intraperitoneal injection. At day 0 and day 3 after infection, sera from the ME49 *Δldh*-vaccinated mice were collected 160 days post-immunization and administered into infected mice by tail vein injection. The protection of passive immunization was estimated in two ways, parasite burden and mice survival. One week post-infection, parasite burden in peritoneal fluid was determined by quantitative PCR. The results showed that passive immunization with the sera of *Δldh*-vaccinated mice did result in significantly lower level of parasite burden compared to immunization with sera of naïve mice (Figure 7A). However, when the survival of mice were followed, passively immunized mice only survived for two more days, compared with the negative immunization control (Figure 7B). Together these results suggested that the sera of *Δldh*-vaccinated mice are able to reduce parasite propagation to some degree but do not offer full protection against lethal infections.

### Robust Pro-Inflammatory Cytokine Production by Splenocytes of *Δldh*-Vaccinated Mice Upon *T. gondii* Antigen Stimulation

**Figure 8:**
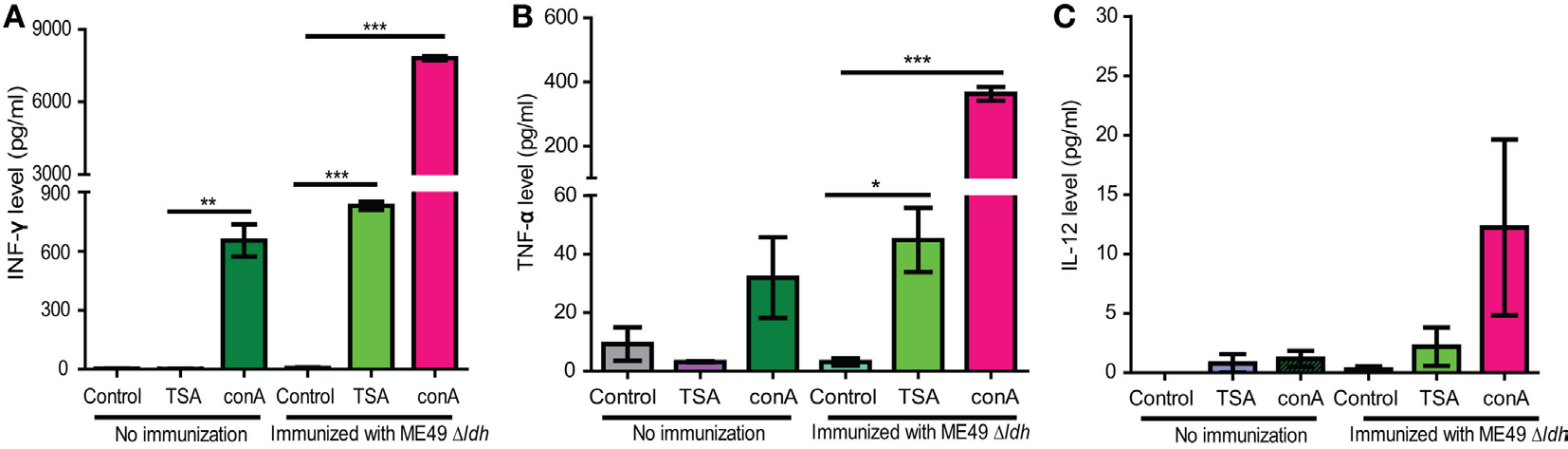
Cytokine production by splenocytes of ME49 *Δldh*-vaccinated mice after *Toxoplasma* antigen stimulation. ICR mice were vaccinated with 10^3^ tachyzoites of ME49 *Δldh* and splenocytes were harvested 160 days post-vaccination. The *in vitro* cultured splenocytes were then treated with fetal bovine serum (control), total soluble Toxoplasma antigen (TSA) or Concanavalin A (conA, positive control) for 72 h. Subsequently the levels of IFN-γ **(A)**, TNF-α **(B)** and IL-12 **(C)** in the culture supernatants were measured by ELISA. Splenocytes isolated from non-immunized naïve mice were included as controls. Three mice from each group were analyzed and each treatment condition was repeated three times, *p < 0.05, **p < 0.01, ***p < 0.0001, Student’s *t*-test.

The above passive immunization results suggested that the contribution of humoral immunity to the protection of *Δldh* vaccination is limited. Next, we estimated cell-mediated immunity, as well as the antigen-specific memory responses by checking the antigen recall response in vaccinated mice. To do that, splenocytes from ME49 *Δldh* immunized mice were harvested 160 days post-vaccination, a time point when the cytokine levels in immunized mice was indistinguishable from that of naïve mice. These splenocytes were cultured *in vitro* and stimulated with total soluble Toxoplasma antigen (TSA) prepared from ME49 tachyzoites. Subsequently the supernatants from the cultures were collected to estimate the cytokine levels by ELISA. Compared with the no-stimulation or non-vaccinated splenocyte controls, TSA stimulated high levels of pro-inflammatory cytokines INF-γ and TNF-α (Figures 8A,B). Induction of IL-12 production was not as obvious (Figure 8C). Given that INF-γ is the key activator of cell-mediated immunity for *T. gondii* clearance, quick and robust INF-γ production from vaccinated splenocytes upon TSA stimulation suggests that efficient cellular immune responses would be activated during secondary infections.

### Compare the Protection Efficiency of *Δldh*-Based Vaccines to That of Uracil Auxotrophic Mutants

**Figure 9:**
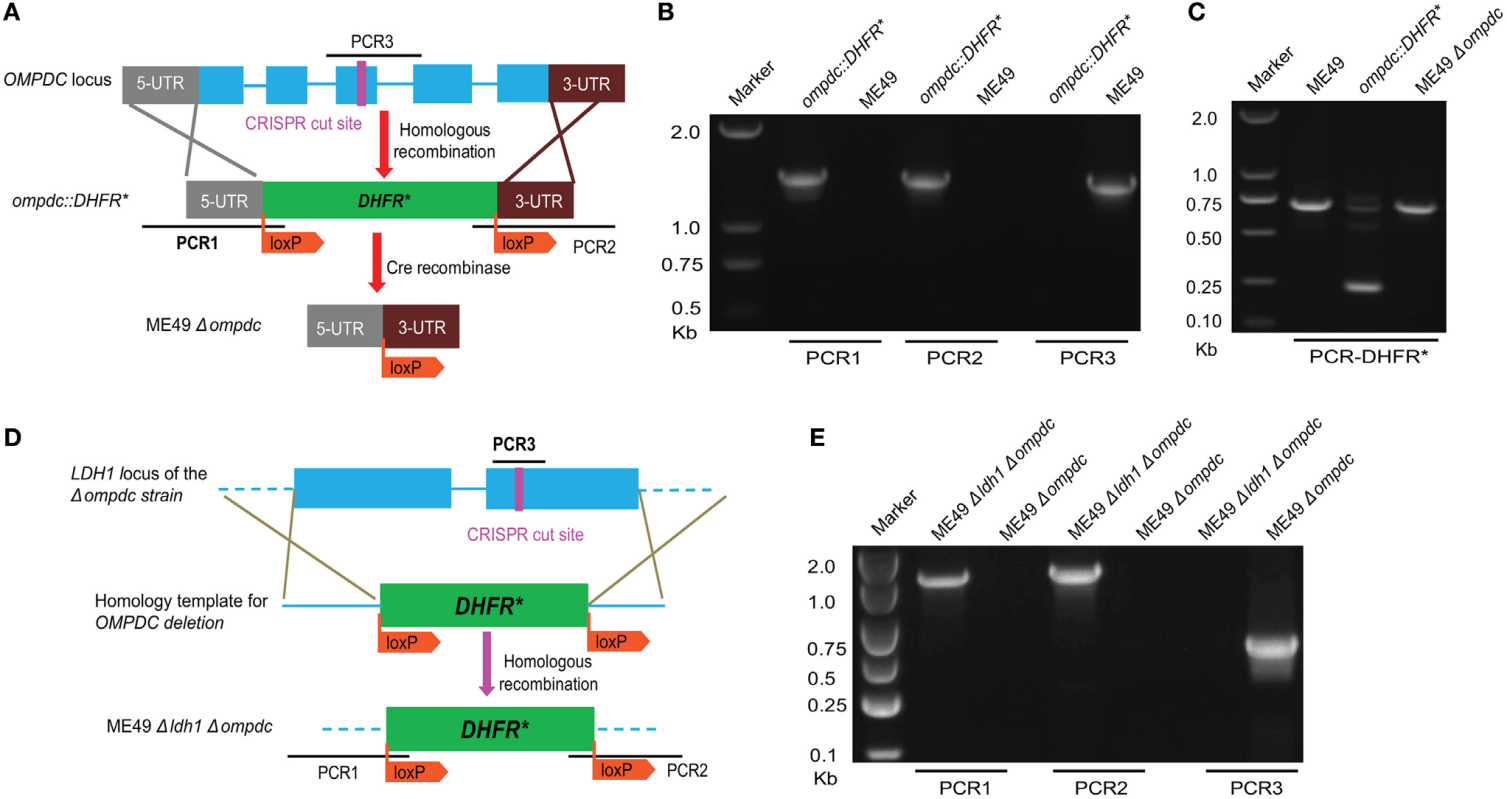
Generation of *OMPDC* deletion mutants. **(A)** Schematic illustration of *OMPDC* replacement by the selection marker DHFR* and subsequent removal of DHFR* by Cre recombinase. **(B)** Diagnostic PCR on an ompdc:DHFR* clone. PCR1 and PCR2 check the integration of DHFR* at the 5′ and 3′ end of *OMPDC*, respectively, whereas PCR3 examines the presence of *OMPDC* sequence. **(C)** Diagnostic PCR confirming the removal of DHFR* by Cre recombinase. This PCR generated a 700 bp product from the endogenous *DHFR* locus, and a 250 bp product from DHFR* due to the absence of introns. Absence of the 250 bp product in ompdc:DHFR* treated with Cre indicates removal of DHFR*. **(D)** Diagram illustrating the deletion of *LDH1* in *Δompdc* to make the double mutant Δompdc ldh1:DHFR*, by CRISPR/Cas9-mediated homologous gene replacement. **(E)** Diagnostic PCR on a Δompdc ldh1:DHFR* clone.

Mutants defective in *de novo* pyrimidine biosynthesis are auxotrophic to uracil, therefore they are not replicating and avirulent *in vivo* (25–27). As such, those types of mutants (such as *CPSII* and *OMPDC* deficient strains) were considered great vaccine candidates. We sought to compare the protection efficiency of our *Δldh*-based vaccines to that based on uracil auxotrophic mutants. For this purpose, we first made an *OMPDC* deletion mutant in ME49, by CRISPR/Cas9-mediated gene replacement to replace *OMPDC* by loxP sites flanked DHFR* (Figure 9A). The resulted ME49 ompdc:DHFR* (Figure 9B) was then transiently transfected with a Cre recombinase expressing plasmid to remove the selection marker DHFR*, to obtain ME49 *Δompdc* (Figure 9C). We also generated a double knockout line (ME49 *Δompdc Δldh1*) by disrupting *LDH1* in ME49 *Δompdc* (Figures 9D,E).

**Figure 10:**
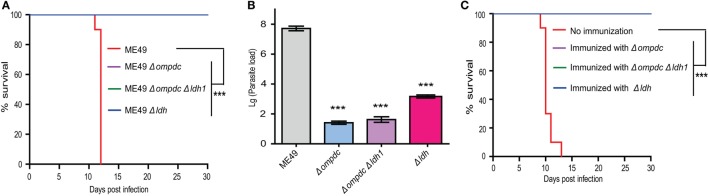
Comparison of *Δldh* mutants-based vaccines to that based on *Δompdc* auxotrophs. **(A)** Survival curves of mice infected with indicated strains. Tachyzoites of indicated strains were used to infect ICR mice (10^4^ parasites/mouse, 10 mice for each strain) by intraperitoneal injection and the survival of mice were monitored for 30 days. ***p < 0.001, Gehan–Breslow–Wilcoxon test. **(B)** Replication of parasites *in vivo*. ICR mice were infected with 10^4^ tachyzoites of indicated strains by intraperitoneal injection and parasite loads in peritoneal fluids 7 days post-infection were determined by quantitative PCR. ***p < 0.001, Student’s *t*-test. **(C)** Protection efficiency offered by vaccination of indicated *Toxoplasma gondii* mutants. ICR mice were immunized with indicated strains by intraperitoneal injection of 10^4^ tachyzoites each. Thirty days post-immunization, they were challenged with 10^4^ tachyzoites of the ME49 strain and survival of mice were monitored for another 30 days. Non-immunized mice were included as negative control. ***p < 0.001, Gehan–Breslow–Wilcoxon test.

First, the virulence of these mutants were compared in mice. With the infection dose of 10^4^ tachyzoites per mouse, ME49 killed all mice within 12 days. All mice infected with *Δompdc, Δompdc Δldh1*, or *Δldh* mutants were survived (Figure 10A), indicating significant virulence attenuation of these mutants. To further check the ability of these mutants to propagate *in vivo*, mice were infected with each mutant line (10^4^ tachyzoites/mouse) and a week later, the parasite burden in peritoneal fluid was examined by qPCR. ME49 displayed robust replication and high parasite burden was detected in mice with this strain (Figure 10B). All three mutants showed minimal replication in mice, as the parasite burden in the corresponding mutants were very low, close to (*Δldh*) or below (*Δompdc, Δompdc Δldh1*) the reliable detection limit of qPCR (Figure 10B). It seemed like there were more parasites in *Δldh*-infected mice than *Δompdc*- and *Δompdc Δldh1*-infected ones, however, because of the sensitivity of the qPCR tests, it is hard to measure the exact parasite burden differences. Next, we compared the immune protection efficiencies of these mutants. Mice were first infected with each of these mutant lines, 30 days post-infection they were challenged with the WT strain ME49 and survival of the mice was monitored. All immunized mice survived the secondary infection (Figure 10C), which is consistent with previous results and indicates that these mutants offered similar protection efficiencies against further infections. Together, these results suggest that the *Δldh* mutant worked comparably well as the uracil auxotrophic mutants as a vaccine candidate.

## Discussion

Decades of epidemiological and experimental studies have suggested that vaccination is probably the most effective way to control the ubiquitous pathogen *T. gondii*, and live attenuated vaccines hold the most promise among all vaccination strategies tried so far. Toxovax^®^ derived from the incomplete strain S48 has been commercialized to reduce *Toxoplasma* caused abortion in sheep (23), and the uracil auxotroph mutants also look promising under experimental settings (25–27). However, these do not mean that current vaccines are ideal. In this study, we tested the possibility of using a lactate dehydrogenase null mutant (*Δldh*) as a live vaccine candidate to prevent animal toxoplasmosis, since our previous work has shown that this mutant was able to grow efficiently *in vitro* but unable to propagate *in vivo* (28). The results showed that, indeed ME49 *Δldh* immunization stimulated efficient protective immunity against the challenge of a variety of strains.

There is a long history for the search of good anti-toxoplasmic vaccines. Killed parasites, total antigens or secreted antigens extracted from cultured parasites, recombinant antigens in a variety of forms, and live attenuated parasites have all been tried over the last 60 years (16, 29–31). One important lesson from these studies is that live attenuated vaccines are the most efficient in stimulating protective immunity. The only commercially available *Toxoplasma* vaccine Toxovax^®^ is a live vaccine derived from the S48 strain, which is used in breeding ewes to reduce incidences of dry ewes and abortion (23). Inability to complete its life cycle was thought to be why it can be used as a vaccine, since it can only grow as tachyzoites and survive in sheep for about 14 days before being cleared by sheep’s immune functions (32). However, this strain is still highly virulent in mice during acute infection. It may also revert to gain cysts or oocysts formation abilities. Because of these limitations, this vaccine is not widely used. Recently the *de novo* pyrimidine synthesis pathway was found to be a good target for vaccine design, as mutants (such as *ΔcpsII* and *Δompdc*) with defective *de novo* UMP biosynthesis activity were able to grow *in vitro* in the presence of uracil but unable to propagate *in vivo* (25–27). Both *ΔcpsII* and *Δompdc* mutants were reported to induce long-term protection against both acute and chronic toxoplasmosis. Although the uracil auxotroph mutants are promising, their protection is not always 100% (25–27). The *Δldh* mutant tested in this study is similar to the uracil auxotroph mutants as a vaccine candidate, due to its drastic growth differences between *in vitro* and *in vivo* conditions.

An ideal live vaccine not only stimulates efficient protection against further infections but also leaves no parasites behind in order to prevent the risk of parasite dissemination through vaccination, particularly for meat producing animals. In the mouse model, *Δldh* vaccination induced strong protective immune responses against the challenge of a variety of strains, including type 1, 2, 3 strains and natural isolates belonging to ToxoDB #9 and ToxoDB #3. However, it should be noted that the protection to virulent type 1 strain RH-Luc is limited to short term, likely because our *Δldh* was made in the type 2 strain ME49. It was reported previously that vaccinated hosts can also be super-infected by wild strains if they contain virulent ROP5 and ROP18 alleles (14). This explains why ME49 *Δldh* vaccination only provided short-term protection against RH infection. It also suggests that using the endemic strains as starting material to design live vaccines is more likely to be successful. Nonetheless, inactivating *LDH* genes is still a feasible way of producing live attenuated vaccines. The other seemingly caveat of ME49 *Δldh* vaccination is that it still produced very small amount of tissue cysts, although we have shown that these cysts were severely attenuated and likely would not cause diseases in vaccinated animals or new hosts if transmitted to. Nevertheless, we have to admit that, ME49 *Δldh* still needs improvement in this regard, especially for meat producing animals due to the risk of transmitting the infection to humans. One strategy to improve, like we showed, is to combine *Δldh* with mutations that lead to defective *de novo* pyrimidine synthesis. The *Δompdc Δldh1* mutant is as efficient as the *Δldh* mutant in terms of stimulating protective immunity against further infections. Yet, there was little parasite replication and no cyst formation *in vivo* after vaccinating animals with this strain (Figure 10B and data not shown), making it safer than the *Δldh* mutant. There is another advantage to combine multiple deletions in one strain for live vaccine design. If the vaccine were to be used in cats, combination of multiple mutations reduces the chance of reversing the vaccine strain back to the virulent parental strain through sexual reproduction, which can happen when the cats are infected with other strains during the vaccination period.

In efforts to assess the potential mechanism of protective immunity from *Δldh* vaccination, we found that it was a combination of humoral and cellular immune responses, with cellular immunity being the major contributor. *Δldh* administration stimulated high levels of IL-12 and IFN-γ, two pro-inflammatory cytokines known to be crucial for activation of cell-mediated clearance of *T. gondii*. The elevated levels of IL-12 and IFN-γ did stay for a while after vaccination and then gradually decreased. They were completely back to normal levels 125 days post-vaccination. At day 160 post *Δldh* immunization, splenocytes extracted from vaccinated mice were able to respond to *T. gondii* antigen quickly and produce high levels of IFN-γ and other pro-inflammatory cytokines, which could activate cellular immune responses and clear secondary infections efficiently. On the other hand, when testing the contribution of humoral immunity by passive immunization, it was found that sera from *Δldh*-vaccinated mice was able to reduce parasite propagation but the overall protection was rather limited, since the passively immunized mice only survived 2 days longer compared to controls (Figure 7B). Together these results suggested that cellular immunity, instead of humoral immunity is the main source of protection from *Δldh* vaccination, similar to the uracil auxotroph mutants-based vaccines (25, 27). In our cytokine measurement studies, we noticed that *Δldh* immunization induced relatively long duration of increased IL-12 and IFN-γ levels (Figures 6A–D). This was somewhat unexpected, but it may be caused by the unique propagation dynamics of the *Δldh* mutants *in vivo*. As was shown, after immunization *Δldh* parasites could form very low levels of cysts in mice, which suggested that some degree of parasite replication and differentiation must have occurred. The prolonged IL-12 and IFN-γ induction might be a consequence of these parasite activities, but further work is required to completely understand this observation. In addition, it is interesting to note that *Δldh* immunization provided full protection against the challenge of type 1 virulent strain in a relatively short term (30 days). If the challenge occurred too long (>75 days) after vaccination, the protection efficiency decreased significantly. This pattern correlated well with the IL-12 and IFN-γ level changes, which may indicate that the existing of an activated cell-mediated immunity is required for full control of virulent type 1 strains. Nonetheless, even after 75 days, vaccinated mice were still partially protected against RH *Δhxgprt* infection, and fully protected from the challenge of other strain types with intermediate virulence.

In conclusion, our study demonstrated that *Δldh* mutant can potentially be used as a live attenuated vaccine. Its administration stimulated a combination of humoral and cellular immune responses that protected animals from further infections of a variety of *T. gondii* strains. We also combined a uracil auxotroph conferring mutation with *Δldh* deletion to make the double knockout *Δompdc Δldh1*, which exhibited similar protection efficiency as the *Δldh* mutant but with further reduced *in vivo* replication and cyst formation, making it safer to use as vaccines. Although the work from mice models demonstrated the great potential of these mutants as vaccine candidates, further investigations are needed to check the safety of vaccination and efficiency of protection in large animals like pigs and sheep, as well as in cats to see the protection against oocyst shedding.

## Materials and Methods

### Mice and Parasite Strains

All 7-week-old ICR mice were purchased from the Hubei provincial center for disease control and prevention. All animals were maintained under standard conditions according to the regulations specified by Administration of Affairs Concerning Experimental Animals. Animal experiments were approved by the ethical committee of Huazhong Agricultural University (permit #: HZAUMO2016049).

*Toxoplasma gondii* type 1 strain RH *Δhxgprt* and its derivative RH-Luc that expresses a firefly luciferase, type 2 strain ME49, type 3 strain VEG, RFLP genotype ToxoDB #3 strain TgPIG-WH1, and ToxoDB #9 strain C7719 were used in this study, which were propagated with human foreskin fibroblast cells (purchased from ATCC, USA) and cultured in DMEM medium supplemented with 10% fetal bovine serum (FBS) (LifeTechnologies, Inc., Rockville, MD, USA). In addition, the *Δompdc* and *Δompdc Δldh1* mutant strains were maintained in the presence of extra 250 µM uracil (25, 27) (LifeTechnologies, Inc., Rockville, MD, USA).

### Virulence Test of *Δldh* Cysts

Fresh brain cysts derived from ME49 *Δldh* or ME49 were used to infect 7-week-old female ICR mice through oral administration (50 cysts/mouse). Mice survival was monitored for 40 days and blood sample were collected afterward. MIC3-based indirect ELISA was used to check the sera to confirm infection. Sero-negative mice were not included in analysis. Cumulative mortality was graphed as Kaplan–Meier survival plots and analyzed in Prism 5 (GraphPad Software, Inc., La Jolla, CA, USA).

### Immune Protection Against Acute Infection Stimulated by *T. gondii* Mutants Vaccination

ICR mice were immunized with 10^4^ tachyzoites of ME49 *Δldh*, ME49 *Δompdc*, or ME49 *Δompdc Δldh1* through intraperitoneal injection. 30, 75, or 125 days post-immunization, mice were challenged with 10^4^ tachyzoites of RH *Δhxgprt*, ME49, VEG, C7719, or TgPIG-WH1 by intraperitoneal injection (10 mice for each group), or 50 fresh cysts of ME49 by oral administration. Non-immunized naïve mice with the same challenging doses and routes were included as controls and subsequently these animals were monitored for another 30 days to check their clinical symptoms and survival. In addition, naïve or *Δldh* immunized mice were infected with 10^4^ tachyzoites of the luciferase expressing RH-Luc strain by intraperitoneal injection (five mice/group). Propagation of RH-Luc in these mice was monitored 1 and 5 days post-infection, by bioluminescent imaging on the IVIS Spectrum imaging system (PerkinElmer, Inc., Boston, MA, USA). To do this, each mouse was injected with 200 µl of 15.4 mg/ml d-luciferin, anesthetized in a chamber containing 2% isoflurane and then imaged by IVIS Spectrum according to the manufacturer’s instructions, as well as previous descriptions (33). Data acquisition and analysis were performed using the Living Image 4 software (PerkinElmer, Inc., Boston, MA, USA).

### Prevention of Cyst Formation by *Δldh* Immunization

ICR mice were first immunized with ME49 *Δldh* as above and then challenged with 10^4^ tachyzoites of TgPIG-WH1. Subsequently the mice were monitored for another 30 days and seropositive mice were sacrificed to harvest the brains. Immunized but not TgPIG-WH1 challenged, and non-immunized but TgPIG-WH1 challenged mice were included as controls (10 mice per group). The number of *Toxoplasma* cysts in each mouse brain was determined by DBA-FITC staining, as previously described (34).

### Cytokines and *Toxoplasma*-Specific IgG Level Measurements

Sera samples were collected from mice and stored at −20°C until analysis. The levels of IL12p70, INF-γ, IL-10, and TNF-α were measured using commercial ELISA kits following the manufacturer’s instructions (4A Biotech, Inc., Beijing, China). To analyze the levels of *T. gondii* specific IgG by ELISA, 96-well microtiter plates were coated with 100 μl/well 0.5 µg/ml soluble tachyzoite antigens diluted in PBS and incubated at 4°C overnight. The plates were then washed and blocked with 1% BSA. Sera samples were diluted 50-fold and added to each well to incubate for 60 min at 37°C. After extensive washing, the bound IgG antibodies were detected by HRP conjugated goat anti-mouse IgG secondary antibodies and tetramethylbenzidine as substrate. A minimum of three mice was included for each experiment and all sera samples were analyzed three times.

### Cytokine Production by Splenocytes After *T. gondii* Antigen Stimulation

ICR mice were immunized with ME49 *Δldh* tachyzoites and seropositive animals were sacrificed 160 days after immunization. Subsequently the spleens were collected from the mice and homogenized in red cell lysis solution (Biosharp, Inc., Beijing, China) to obtain splenocyte suspensions. The recovered splenocytes were washed once in RPMI 1640 (LifeTechnologies, Inc., Rockville, MD, USA), resuspended and counted (35). Viability of the cells was determined by trypan blue staining. Then 3 × 10^5^ viable splenocytes were plated in each well of 96-well plates and cultured in RPMI 1640 supplemented with 20% FBS (LifeTechnologies, Inc., Rockville, MD, USA) and 100 U/ml penicillin–streptomycin. Meanwhile the splenocytes were stimulated with total *T. gondii* soluble antigens (final concentration 50 µg/ml) isolated from tachyzoites of ME49 for 3 days. The supernatants from these cultures were harvested for cytokines level measurements, which was performed as above. *T. gondii* soluble antigens were prepared as the following: freshly egressed tachyzoites of ME49 were collected, purified by filtration, and lysed by sonication in a waterbath sonicator for 30 min. Subsequently the samples were spinned at 12,000 rcf for 10 min at 4°C and the supernatant was collected as TSA, which was subject to protein concentration determination by BCA Protein Assay Kit (Beyotime Biotechnology, Beijing, China) before use. The splenocytes were also stimulated with 5 µg/ml concanavalin A (LifeTechnologies, Inc., Rockville, MD, USA) or 20% FBS as positive and negative controls, respectively. The same experiments were also done with non-immunized naïve mice for further comparisons. Three naïve mice and three immunized mice were analyzed, and each supernatant sample was examined three times by ELISA for technical replication.

### Passive Immunization With the Sera of *Δldh*-Vaccinated Mice

ICR mice (16 mice in total, two groups with 8 mice in each group) were infected with the WT strain ME49 through intraperitoneal injection (10^4^ tachyzoites/mouse). At day 0 and day 3 after infection, sera from naïve mice or the ME49 *Δldh*-vaccinated mice 160 days post-immunization were collected and administered into infected mice by tail vein injection (100 μl/mouse). The protection of passive immunization was estimated in two ways. First, three mice from each group were sacrificed 7 days post-infection to measure the parasite burden in peritoneal fluids by quantitative PCR, as previously described (28). Second, the survival of the other five mice in each group was monitored daily for 30 days.

### Generation of *Δompdc* and *Δompdc Δldh1* Mutants

Both *OMPDC* and *LDH1* deletions were made by CRISPR/Cas9-mediated homologous gene replacements, using the methods described previously (36, 37). All primers and plasmids used in the mutant constructions are listed in Tables 1 and 2, respectively. *LDH1* and *OMPDC* specific CRISPR plasmids were generated by replacing the *UPRT* targeting guide RNA (gRNA) in *pSAG1-Cas9-sgUPRT* with corresponding gRNAs, using Q5 site-directed mutagenesis (36, 37). The plasmid p*OMPDC*:loxp-DHFR*-loxp was constructed by cloning the 5′- and 3′-homology arms (1,044 and 981 bps, respectively) of *OMPDC*, as well as the loxp-DHFR*-loxp (3,672 bps) amplified from pDONR-G265-loxP-DHFR*-loxp into pUC19 through Gibson assembly (New England Biolabs, Ipswich, MA, USA). It was used as the homologous template to replace *OMPDC* with the loxp-DHFR*-loxp. The plasmid pLDH1:loxp-DHFR*-loxp used to replace *LDH1* with loxp-DHFR*-loxp was constructed in a similar way. All the plasmids were verified by DNA sequencing before use. To constructed the *OMPDC* knockout strain, ME49 was co-transfected with the *OMPDC* specific CRISPR plasmid and homologous template (*OMPDC*:loxp-DHFR*-loxp), selected with 1 µM pyrimethamine and 250 µM uracil (LifeTechnologies, Inc., Rockville, MD, USA), and single cloned by limiting dilution. The resulted ME49 ompdc:DHFR* was then transiently transfected with a Cre recombinase expressing plasmid (pmin-Cre-eGFP) (38) to remove the selection marker DHFR*, to obtain ME49 *Δompdc*. All clones were identified by diagnostic PCRs, as demonstrated in Figures 9A,D. The *Δompdc Δldh1* double mutant were made by replacing *LDH1* in ME49 *Δompdc* with loxp-DHFR*-loxp, using the same strategy as the *OMPDC* deletion.

**Table 1:** Primers used in this study.

Primer	Sequence	Use
gRNA-LDH1-Fw	5′-GCCGGTCTGACCAAGGTGCCGTTTTAGAGCTAGAAATAGC-3′	To construct the *LDH1* specific CRISPR plasmid
gRNA-OMPDC-Fw	5′-GAGCTTGACTCCACCCTCACGTTTTAGAGCTAGAAATAGC-3′	To construct the *OMPDC* specific CRISPR plasmid
gRNA-R	5′-AACTTGACATCCCCATTTAC-3′	To construct gene specific CRISPR plasmids
pUC19-Fw	5′-GGCGTAATCATGGTCATAGC-3′	Amplification of pUC19 for Gibson assembly
pUC19-Rv	5′-CTCGAATTCACTGGCCGTCG-3′	Amplification of pUC19 for Gibson assembly
loxp-DHFR*-loxp-Fw	5′-CAACCCGCGCAGAAGACATC-3′	Amplification of loxp-DHFR*-loxp for Gibson assembly
loxp-DHFR*-loxp-Rv	5′-GGACACGCTGAACTTGTGGC-3′	Amplification of loxp-DHFR*-loxp for Gibson assembly
U5ompdc-Fw	5′-CGACGGCCAGTGAATTCGAGGTACGTTTGCGATTGTGAGC-3′	Amplification of 5′-homology of *OMPDC* for pOMPDC:loxp-DHFR*-loxp construction
U5ompdc-Rv	5′-GATGTCTTCTGCGCGGGTTGCAGTTCTTTGGAATTGTCACGG-3′	Amplification of 5′-homology of *OMPDC* for pOMPDC:loxp-DHFR*-loxp construction
U3ompdc-Fw	5′-GCCACAAGTTCAGCGTGTCCCGTGCTGAAAGCGAAACACTATC-3′	Amplification of 3′-homology of *OMPDC* for pOMPDC:loxp-DHFR*-loxp construction
U3ompdc-Rv	5′-GCTATGACCATGATTACGCCGTCAGTTTCTCTGTGCGAGTTG-3′	Amplification of 3′-homology of *OMPDC* for pOMPDC:loxp-DHFR*-loxp construction
U5ldh1-Rv	5′-GATGTCTTCTGCGCGGGTTGTTCTCCTCTGCACAAGTGCG-3′	Amplification of *PUC19:LDH1-5UTR-3UTR* for pLDH1: loxp-DHFR*-loxp construction
U3ldh1-Fw	5′-GCCACAAGTTCAGCGTGTCCTGGCAAAACAGGAGCGGAAT-3′	Amplification of *PUC19:LDH1-5UTR-3UTR* for pLDH1: loxp-DHFR*-loxp construction
5′-UpU5OMPDC	5′-GACAGTTATGGCCCGTCTTC-3′	PCR1 of Δompdc:DHFR*
3′-InDHFR*-Fw	5′-CGTGACCACGCCAAAGTAG-3′	PCR1 of Δompdc:DHFR*
5′-InDHFR*-Rv	5′-GCACTTGCAGGATGAATTCC-3′	PCR2 of Δompdc:DHFR*
3′-DnU3OMPDC	5′-GAGCATTGTGCATTTGCGTC-3′	PCR2 of Δompdc:DHFR*
5′-UpgRNA OMPDC	5′-CGTCAGCTTCGTTAGACCAG-3′	PCR3 of Δompdc:DHFR*
3′-DngRNA OMPDC	5′-CAGCTGTACTATGAACGGGTG-3′	PCR3 of Δompdc:DHFR*
5′-In*DHFR*	5′-GTCCGAGTCTGTGCTTCAC-3′	To identify the deletion of loxP-DHFR*
3′-In*DHFR*	5′-CCTGCGCAGCAGTTGATTTG-3′	To identify the deletion of loxP-DHFR*
5′-InU5ldh1	5′-CTGTCCAGCGTAGCAATCAC-3′	PCR1 of *Δompdc Δldh1*
3′-In DHFR*	5′-ACCACGCCAAAGTAGAAAGG-3′	PCR1 of *Δompdc Δldh1*
5′-InDHFR*	5′-TGCTGGACTGTTGCTGTCTG-3′	PCR2 of *Δompdc Δldh1*
3′-InU3ldh1	5′-GTGGCCGACCTTTTCAGAGC-3′	PCR2 of *Δompdc Δldh1*
5′-UpgRNA ldh1	5′-GCTAGACGCGGAGAGTTGTG-3′	PCR3 of *Δompdc Δldh1*
3′-DngRNA ldh1	5′-AGCTGCTTCTCCGTGACTAC-3′	PCR3 of *Δompdc Δldh1*
RT-tubulin-F	5′-CACTGGTACACGGGTGAAGGT-3′	β-tubulin-based qPCR
RT-tubulin-R	5′-ATTCTCCCTCTTCCTCTGCG-3′	β-tubulin-based qPCR

**Table 2:** Plasmids used in this study.

Plasmid name	Use	Source
pSAG1-Cas9-sgUPRT	Template for gene-specific CRISPR plasmid construction	Addgene plasmid #54467
pSAG1-Cas9-sgLDH1	LDH1-specific CRISPR plasmid	(28)
pSAG1-Cas9-sgOMPDC	OMPDC-specific CRISPR plasmid	This work
pOMPDC: loxp-DHFR*-loxp	To replace OMPDC with loxp-DHFR*-loxp	This work
pLDH1: loxp-DHFR*-loxp	To replace LDH1 with loxp-DHFR*-loxp	This work
pLDH1:*DHFR*	Template for *PUC19:LDH1-5UTR-3UTR* amplification	(28)
pDONR-G265-loxP-DHFR*-loxp	Template for loxp-DHFR*-loxp amplification	This work
pmin-Cre-eGFP	To remove the selection marker DHFR*	(38)

### Statistical Analysis

Statistical comparisons were performed in Prism 5 (GraphPad Software, Inc., La Jolla, CA, USA) using Student’s *t*-tests, Gehan–Breslow–Wilcoxon test or one-way ANOVA with Bonferroni posttests as indicated in figure legends.

## Ethics Statement

All animals were maintained under standard conditions according to the regulations specified by Administration of Affairs Concerning Experimental Animals. Animal experiments were approved by the ethical committee of Huazhong Agricultural University (permit #: HZAUMO2016049).

## Author Contributions

BS, NX, and TZ conceived and designed the experiments; NX, TZ, XL, SY, PZ, and JY performed the experiments; BS, NX, TZ, YZ, and JZ analyzed the data; BS and NX wrote the paper.

## Conflict of Interest Statement

The authors declare that the research was conducted in the absence of any commercial or financial relationships that could be construed as a potential conflict of interest.
